# Dietary Intake of Multiple Nutrient Elements and Associated Health Effects in the Chinese General Population from a Total Diet Study

**DOI:** 10.3390/nu15112613

**Published:** 2023-06-02

**Authors:** Lan Ma, Huijing Shen, Xiaohong Shang, Shuang Zhou, Bing Lyu, Xin Zhao, Jingguang Li, Yunfeng Zhao, Yongning Wu

**Affiliations:** 1 NHC Key Laboratory of Food Safety Risk Assessment, China National Center for Food Safety Risk Assessment, Beijing 100022, China; malan@cfsa.net.cn (L.M.); 2250021020@st.btbu.edu.cn (H.S.); lvbing@cfsa.net.cn (B.L.); lijg@cfsa.net.cn (J.L.); zhaoyf@cfsa.net.cn (Y.Z.); wuyongning@cfsa.net.cn (Y.W.); 2 School of Food and Health, Beijing Technology and Business University, No. 11 Fu-Cheng Road, Beijing 100048, China; 3 Food Safety Research Unit (2019RU014) of Chinese Academy of Medical Science, China National Center for Food Safety Risk Assessment, Beijing 100021, China; shangxh@cfsa.net.cn

**Keywords:** nutrient elements, total diet study, dietary intake, regional distribution, food sources, health effect

## Abstract

Nutrient elements are essential for human health. The intake of nutrient elements (Na, K, Ca, Mg, P, Mn, Fe, Zn, Cu, Se, Mo, and Cr) in the general Chinese population was comprehensively evaluated via a recent total diet study (2016–2019), covering more than two-thirds of the total population. The contents of nutrient elements in 288 composite dietary samples were determined by ICP-MS. The dietary sources, regional distribution, the relationship with the earth’s crust, dietary intake, and health effects were discussed. Plant foods were the main source of both macro-elements and trace elements, accounting for 68–96% of total intake. Trace elements in food were compatible with their abundance in the earth’s crust. Na intake reduced by 1/4 over the last decade but was still high. The average intake of Ca, Mg, Zn, and Se did not reach the health guidance values, while the average intake of K, P, Mn, Fe, Cu, Mo, and Cr fell within a reasonable range. No element exceeded the UL. However, an imbalance was identified in the dietary Na/K ratio and Ca/P ratio. This paper provides a most recent and national-representative assessment of nutrient element intake, indicating the significance of salt reduction and dietary structure optimization for the population.

## 1. Introduction

Human tissues contain various natural elements, more than 20 of which are critical to support body metabolism and the maintenance of physiological functions [1]. These elements regulate the permeability of cell membranes to maintain the concentration of inorganic ions in intracellular and extracellular fluids. They also support normal osmotic pressure of intracellular and extracellular fluids and help to maintain an optimal acid–base balance in the body. Insufficient nutrient intake by the human body will have an adverse effect on metabolic processes, and long-term insufficient intake can also cause subclinical deficiencies and even nutritional deficiency diseases, such as growth retardation in children, Fe-deficiency anemia, osteoporosis, Keshan disease, and other diseases [1,2].

K, Na, Ca, Mg, and P in the human body with contents greater than 0.01% are called macro-elements. Elements with contents less than 0.01% in the human body, such as Fe, Cu, Zn, Cr, Mn, Se, etc., are called trace elements, and are necessary for the human body. These trace elements are involved in the body’s metabolism, but cannot be produced and synthesized by the body, and must be continuously ingested and supplied by food. Therefore, it is necessary to ensure diet quality and the intake of trace elements [3,4]. 

Nutrient elements are closely related to human health. Excessive or insufficient nutrient intake may contribute to abnormal human physiology or disease. Although required in small amounts in the body, therapeutic and toxic doses of trace elements have a narrow range, and excessive intake of some elements can poison the human body. For example, high dietary Se intake can cause chronic and acute poisoning [5]. Fe itself is not toxic, but excessive Fe intake may cause Fe toxicity, and severe cases can lead to diseases such as diabetes, heart failure, and cancer [6]. Excess or deficiency of Zn and Cu also cause adverse effects [4,7,8]. Mo levels in food and water exceeding 100 mg/kg can cause symptoms of toxicity [2,7]. In this regard, the WHO and some countries have set guidance values for the intake of nutrient elements, as summarized in Table S1.

Elements are key components of human tissues and are critical to maintain the excitability of nerves and muscles. The distribution of elements in the body is extremely imbalanced. Ca, P, and Mg are mainly distributed in bones and teeth, and Fe is distributed in red blood cells, a component of hemoglobin, whereas Zn is distributed in muscle tissue. Ca is an essential element for normal nerve impulse transmission. Ca, Mg, and K have important regulatory effects on muscle contraction and relaxation. Various elements in food act synergistically or antagonistically with each other. For example, an inappropriate ratio of Ca and P in the diet can affect the absorption of these two elements, such that excess Mg interferes with Ca metabolism, excess Zn affects Cu metabolism, and excess Cu can inhibit Fe absorption. Elements are also components of hormones, vitamins, proteins, and various enzymes [3]: glutathione peroxidase contains Se and Zn, and cytochrome oxidase contains Fe and other elements [1].

A total diet study (TDS), also known as a market basket study, is an assessment of the dietary intake of food nutrients/contaminants in a population or subpopulation representing the dietary habits [9]. It measures the intake of various dietary chemical constituents (contaminants, nutrients) by the residents of a country or region through cooked and ready-to-eat diets (including drinking water), as well as the possible health risks posed by the intake of these substances. The TDS is considered as the most efficient and cost-effective method for assessing dietary nutrient intake in large populations and has been used in many countries around the world. China has successfully conducted six TDSs since 1990 [10,11] (pp. 64–88, pp. 94–118). A comprehensive method and system with Chinese characteristics have been established after nearly three decades of development and evolution.

During 2016–2020, the latest China TDS (the 6th) was conducted, including a dietary survey, food aggregation, collection of individual samples, cooking and preparation of food composite samples, experimental analysis of food samples, and a dietary exposure assessment. The geographical areas involved in this new round of the China TDS were further enlarged to 24 provinces, representing the dietary habits of various regions of China and covering over 2/3 of the population. In this paper, a total of 288 composite dietary samples from the 6th China TDS were analyzed using inductively coupled plasma-mass spectrometry (ICP-MS) to investigate the nutrient elements, Na, K, Ca, Mg, P, Mn, Fe, Zn, Cu, Se, Mo, and Cr. The dietary sources and regional distribution of these elements, the relationship with the environment, as well as their dietary intake and potential health effects, were analyzed to achieve a most recent and national-representative assessment of the nutrient elements in the Chinese population.

## 2. Materials and Methods

### 2.1. Food Sampling and Food Consumption Data

Compared with the 5th TDS [11], the number of locations involved in the 6th TDS in China increased from 20 provinces (municipalities and autonomous regions) to 24, with Shanxi and Gansu provinces added to the northern region, and Shandong and Guizhou provinces to the southern region. The dietary survey protocol, sample collection, and processing methods were basically the same as those applied in the 5th TDS [11]. Provinces with a population of more than 50 million had 6 survey sites, and provinces with a population of less than 50 million had 3 survey sites, containing 2 rural sites and 1 urban site. Selected rural sites were representative of local dietary habits and areas in the province with a moderate economic status. Urban sites should be selected among small and medium-sized cities. The comprehensive results that were obtained should represent the average dietary composition of the province. A total of 30 households were randomly selected and surveyed at each site. The survey was carried out among all family members by combining weighing and recording methods to collect household food consumption data over a period of three days. The contents included all data related to food and drinking water, as well as the condiments consumed by the respondents during the survey period.

Based on the consumption survey, food consumption per capita was aggregated into 13 categories, consisting of cereals, legumes, potatoes, meat, eggs, aquatic products, dairies, vegetables, fruits, sugars, beverages and water, alcohol, and condiments. The most consumed food items in each food category were collected locally at the survey sites. They were then cooked according to local customs, and the condiments were added to the other 12 food categories during the cooking procedure. Therefore, there were 12 categories of samples for laboratory determination. In each province, the individual cooked samples were homogenized to obtain a composite sample for each food category, then put into polyethylene plastic containers and placed in a low-temperature refrigerator at −20 °C for storage. This study involved 12 food categories from 24 provinces, with a total of 288 composite food samples.

### 2.2. Detection Method, Detection Limit, and Undetected Data Processing

The detection method was based on the China national food safety standard “Determination of Multiple Elements in Foods” (GB 5009.268-2016), and inductively coupled plasma mass spectrometry (ICP-MS) was used to determine the content of elements in the samples. The limit of detection (LOD) of various elements was as follows: Na, 125 μg/kg; K, 80 μg/kg; Ca, 150 μg/kg; Mg, 15 μg/kg; P, 200 μg/kg; Mn, 1 μg/kg; Fe, 8 μg/kg; Zn, 4 μg/kg; Cu, 2 μg/kg; Se, 0.25 μg/kg; Mo, 0.5 μg/kg; Cr, 1 μg/kg. In this study, the detection rates of the 12 elements were all greater than 40%. Therefore, all the results below the detection limit were set as 1/2 LOD for the intake calculation according to the recommendations of the World Health Organization [12].

To enhance the accuracy and reliability of the analysis, the samples were measured in duplicate (represented by the average value of the two measurements), and at the same time, two blanks were performed for each batch of samples to eliminate any interference caused by background contamination of the measurement. In addition, certified reference materials with the same or a similar food matrix were selected as the quality control samples. The measurement results of the quality control samples were consistent with the certified values (Table S2).

### 2.3. Estimation of Dietary Intake

Calculation formula for dietary element intake:

(1) $$\mathrm{DI}=\overset{p}{\underset{k=1}{\sum }}\frac{X_{k}\times C_{k}}{\mathrm{BW}}$$

where *X_k_* expresses the consumption of the *k*-th food, *C_k_* is the content of the element in the *k*-th food, *p* represents the food category (12 categories in this study), and BW is the average body weight of the population (63 kg).

According to the recommended nutrient intake (RNI), adequate intake (AI), and tolerable upper intake level (UL) outlined in “Chinese Dietary Reference Intakes (2013 version)” [13], the intake status and health effects of macro-elements and trace elements in the daily diet of Chinese residents was evaluated by China’s 6th TDS.

## 3. Results and Discussion

### 3.1. Analysis of Nutrient Elements in Dietary Samples

**Figure 1:**
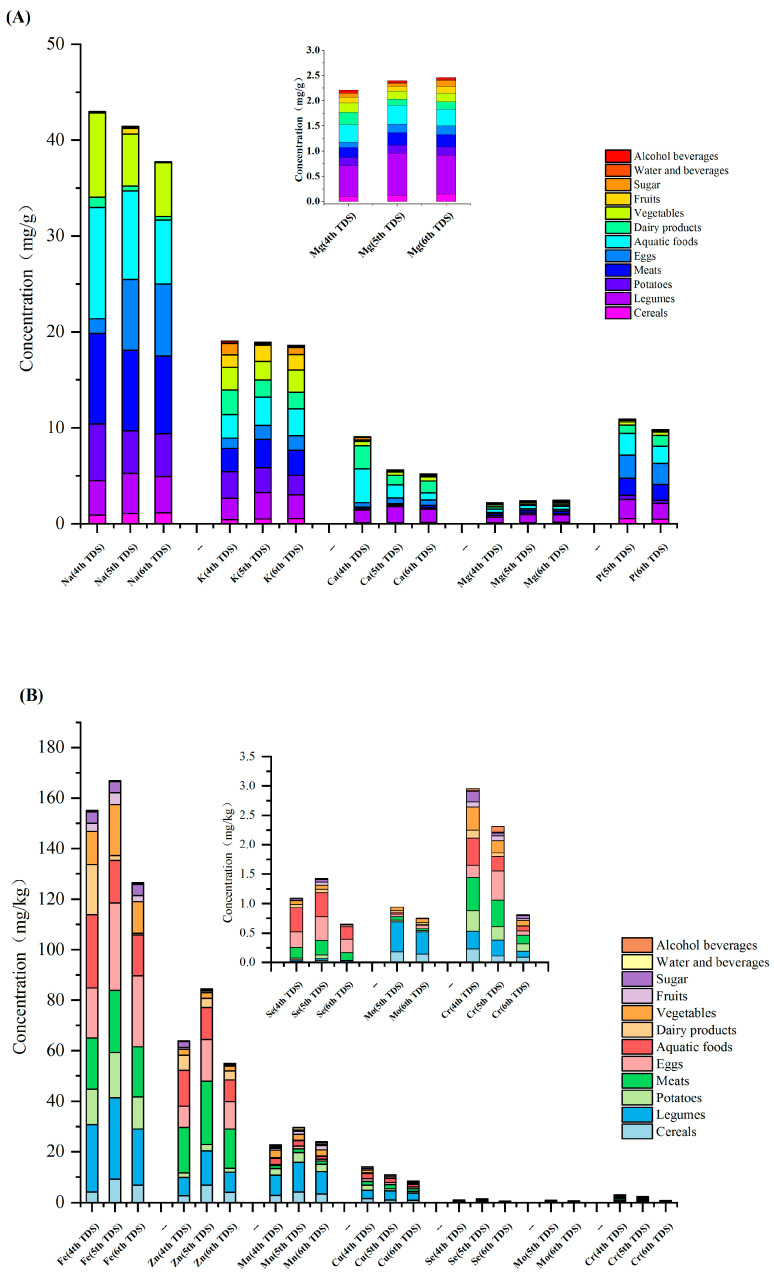
Average concentrations of macro-elements (**A**) and trace elements (**B**) in 12 food categories in the 4th, 5th, and 6th China TDSs.

The detection rates of macro-elements, including Na, K, Ca, and Mg, in 12 categories of dietary composite samples from 24 provinces (municipalities and autonomous regions) in the country were all 100%, and the detection rate of P was 99.7%. The detection rates of trace elements were as follows: Fe (99.3%), Mn (100%), Zn (97.9%), Cu (97.2%), Se (89.2%), Mo (88.5%), and Cr (92.0%). The content determination results of elements in the 12 types of total dietary samples are shown in Figure 1 and Table S3.

#### 3.1.1. Content of Macro-Elements in Dietary Samples

The average content of Na was the highest in meat, followed by eggs, seafood, and vegetables. The highest Na content was in the egg sample from Zhejiang (14.78 mg/g). In the 4th to 6th TDS, it was found that the Na content of cereals, legumes, and eggs had an increasing trend, and the Na contents of the egg samples, as determined by the 5th and 6th TDSs, were nearly three-times higher than that of the 4th TDS, which might be related to changes in the way Chinese residents cook eggs. Due to the diversity of egg consumption, salted duck eggs and preserved eggs might have increased the Na content of the composite egg samples in the 5th and 6th TDS, while the higher consumption of egg soup in the 4th TDS may have significantly reduced the Na content.

Foods containing high levels of K were basically the same as those with high levels of Na, with aquatic products having the highest K content, followed by meat, legumes, and vegetables. The highest K content was found in a legume sample (4.67 mg/g). High contents of K might be due to the fact that plants grow in soil treated by K fertilizer and 0.5% K dihydrogen phosphate solution might be used for foliar spraying. On the other hand, it might be attributed to the cooking process. To improve foods’ taste, salt, soy sauce, spices, and other condiments are often added during cooking. The K content of dietary sugar samples greatly varied. In the 4th to 6th TDSs, the type of food with a high K content did not significantly change.

The Mg content of legumes was the highest, followed by aquatic products and meat. The Mg content of aquatic products was only 42% of that found in legumes, and the Mg content of meat was only 32% of that found in legumes. The results showed that legumes are rich in Mg, which was consistent with the data from the previous two TDSs. Compared with the 4th and 5th TDSs, the Mg content of sugar in the 6th TDS significantly increased, by 68% and 135%, respectively.

The Ca content of legumes was the highest, followed by dairy and aquatic products. Milk and dairy products are not only rich in Ca, but also have a high absorption rate. The categories of total dietary samples with the highest Ca content were consistent across the three TDSs.

The average phosphorous content of eggs was the highest, followed by aquatic products, meat, and legumes. The food types with the highest P content, as determined by the 6th TDS, were consistent with the findings of the 5th TDS. The highest P content (i.e., 3.03 mg/g) was found in an aquatic sample from Liaoning province.

#### 3.1.2. Trace Elements in Dietary Samples

The Fe content of eggs was the highest, followed by legumes and meat. The consumption of these foods is recommended to treat and prevent Fe deficiency and anemia. Compared with the 5th TDS, the 6th TDS showed a decrease in the dietary Fe content, except for alcohol, and the reduction rate ranged from 0.7% (sugar) to 47.5% (fruit). Compared with the 4th TDS, the 6th TDS revealed that the Fe content of cereals increased by 67.2%, whereas the Fe content of aquatic products decreased by up to 45%.

The average Mn content of legumes was the highest, followed by cereals and potatoes. In addition, the Mn content of legumes was nearly three times higher than that found in other food sources. The highest Mn content (15.11 mg/kg) was found in legumes from Liaoning province and the lowest (3.71 mg/kg) was detected in legumes from Ningxia province. The Mn content in the legume samples greatly varied among different provinces. The Mn content of water, beverages, and alcohol showed the most obvious regional differences, amounting to an almost 500-fold difference. The Mn content of cereal samples showed the minimal regional differences, with the highest value in Henan province (4.49 mg/kg) being 2.1 times higher than that of Gansu province, where the lowest level was detected (2.11 mg/kg). However, Mn rarely causes poisoning, and studies have shown that the main clinical cases of Mn poisoning were from industrial occupational Mn exposure [14]. In general, a diet of meat and vegetables can basically meet human requirements for Mn. Comparing the results of the 4th, 5th, and 6th TDSs, it was found that the Mn content of legumes was 25% lower in the 6th TDS. Moreover, compared with the findings of the 4th and 5th TDSs, the Mn content of cereals in the 6th TDS decreased by 54% and 19%, respectively.

The average content of Zn was the highest in meat, followed by eggs, aquatic products, and legumes. The highest Zn content was detected in the aquatic sample from Liaoning province (22.51 mg/g). Compared with the results of the 4th and 5th TDSs, the 6th TDS revealed that Zn content showed an overall downward trend.

The Cu content of legumes was the highest, followed by aquatic and meat products. The Cu content of legumes was more than twice that detected in aquatic and meat products. Both the results of the 4th and 5th TDSs showed that the Cu content of legumes was much higher than that found in other types of food. The data obtained from the 6th TDS showed that the largest decrease of Cu content was detected in vegetables, which contained 59.8% and 22.3% less Cu when compared with the 4th and 5th TDSs, respectively.

The average Se content of eggs was the highest, followed by aquatic and meat products. The average Se content of other dietary samples was only 10% of that detected in eggs. The egg sample from Jiangxi province had the highest Se content (410.21 μg/kg). Se content showed a significant downward trend in all kinds of samples analyzed by the 6th TDS, with cereals and aquatic products decreasing by 49.3% and 47.6%, respectively, compared with the 4th TDS. Furthermore, compared with the 5th TDS, Se content in meat, cereals, aquatic products, and eggs decreased by 43.7% to 71.8%.

The Mo content of legumes was the highest, followed by cereals. Compared with the 5th TDS, except for eggs and milk, the Mo content of other food samples analyzed in the 6th TDS showed a decreasing trend.

The Cr content was higher in meat, followed by potatoes and legumes. Compared with the data obtained in the 5th TDS, the 6th TDS revealed that the Cr content of other samples showed a downward trend, except for sugar, which increased by 11.8%. Compared with the 4th TDS, the Cr content of cereals, vegetables, and meat decreased by 62.2%, 76.8%, and 74.9%, respectively, and compared with the 5th TDS, a respective decrease of 21.0%, 56.9%, and 68.8% was observed.

#### 3.1.3. Food Categories and Regional Distribution Characteristics of Nutrient Elements

The content of macro-elements in cereals, fruits, sugars, water and beverages, and alcohol samples was significantly lower than that in legumes, potatoes, meat, eggs, aquatic products, dairy, and vegetable samples. The content of trace elements in milk, vegetables, fruits, sugars, water and beverages, and alcohol samples was significantly lower than that in cereals, legumes, potatoes, meat, eggs, and aquatic products, as shown in Table 1. It was found that the content of the element was greatly affected by the food categories, food processing, and the cooking process.

Food processing and refining may result in the loss of various elements. For example, rich minerals on grain surfaces are often lost due to excessively fine grinding. The loss of a large amount of water-soluble minerals is caused by soaking vegetables in water or pouring out the water after boiling the vegetables, and by washing the rice too many times [1]. 

There were regional differences in the content of Na, K, P, Ca, Mg, and Fe in the cereal samples of TDSs, and the content of these elements was significantly higher in the northern region than in the southern region. The Cr content of legumes and vegetables grown in northern regions was significantly lower than that in southern regions (Figure S1). No significant regional differences were found in the distribution of other elements. At the same time, it was found that the content of elements was also closely related to the growth environment, especially the soil.

Most minerals come from the earth’s crust [15], but their distribution is imbalanced. Food chains link concentrations of elements in human, food, and environmental media, such as crust or soil [16]. The decreasing order of macro-element concentrations in the crust, upper crust, and soil was as follows: Ca > Na > K > Mg > P [17,18], while the order was: Na > K > P > Ca > Mg in cereals, legumes, vegetables, meat, aquatic products, and potatoes. Concentrations of Na, K, and P in these food categories were consistent with the order observed in crusts and soil, while concentrations of Na and K were greater than those of Ca. Plants and animals can increase the uptake of some elements, so the concentrations and order of elements is likely to differ from the order found in the earth’s crust. In addition, since the dietary samples in the TDS were cooked with condiments, such as salt and soy sauce, this might also increase the Na and K levels in some food categories. In fruits, the order was: K > Mg > P > Ca > Na. Concentrations of K, Mg, and P were consistent with the order detected in crusts and soil, and concentrations of Ca and Na were relatively low. In addition to the enrichment properties of plants for certain elements, the higher concentrations of P and K in fruits than in crusts might also be attributed to the use of P and K fertilizers in plant growth.

The descending order of the concentrations of trace elements in the crust, upper crust, and soil was: Fe > Mn > Zn > Cr > Cu > Mo > Se [17,18]. The order of contents of trace elements in potatoes, vegetables, and fruits was: Fe > Mn > Zn > Cu > Cr > Mo > Se. The order of trace elements’ content in legumes, cereals, meat, and aquatic products was: Fe > Mn > Zn > Cu > Mo > Cr > Se, Fe > Zn > Mn > Cu > Mo > Cr > Se, Fe > Zn > Mn > Cu > Cr > Se > Mo, and Fe > Zn > Mn > Cu > Se > Cr > Mo, respectively. The differences in Cu and Cr contents in these food categories might be due to the selectivity of plants for elemental uptake or the application of a fertilizer. An appropriate amount of nitrogen fertilizer can increase the content of trace elements and improve the nutritional quality [19]. Except for the differences in Cu and Cr contents in potatoes, vegetables, and fruits, the distribution characteristics of other element concentrations were consistent with those in crusts and soil. Differences in Zn and Mn concentrations in cereals, meat, and aquatic products, and Se and Mo concentrations in meat and aquatic products, may be due to differences in the selective enrichment of trace elements by organisms [19,20]. The difference in Mo and Cr concentrations in cereals and legumes might be related with ammonium molybdate, which has been widely used as a trace element fertilizer at home and abroad in recent years, as it can significantly improve the quality and yield of beans, forages, and other crops.

### 3.2. Intake and Dietary Sources

The recommended nutrient intake (RNI), adequate intake (AI), and tolerable upper intake level (UL), as outlined in “Chinese Dietary Reference Intakes (2013 version)” [13], were used to evaluate the dietary nutrient intake of Chinese people. In accordance with the 6th TDS, the intakes of 12 nutrient elements in the average diet of Chinese residents are listed in Table 2. In general, the average dietary intakes of Na, K, Cr, P, Fe, Cu, Mn, and Mo by Chinese residents met the health-based guidance values. The dietary intakes of P, Fe, Cu, and Mo were between the RNI and the UL, and the dietary intake of Mn was between the AI and the UL. The above intakes fell within an appropriate range and did not exceed the maximum tolerable values set by the Chinese Nutrition Society for adults. The average intakes of Ca, Zn, Mg, and Se in the diet have not yet reached the recommended health guidance values, and intake levels are still generally inadequate. It is necessary to increase the consumption of aquatic products, meat, legumes, eggs, and dairy foods, so as to reduce the risks of nutritional deficiency posed to the health of residents. Maintaining a balanced and varied diet that meets the needs of the population for nutrient elements is critical to avoid excessive or inadequate intake of some nutrients and prevent risks to human health and safety.

Compared to the recommended nutrient intakes (Table S1) set by the WHO [21,22] and some other countries [23,24,25,26,27], the dietary Na intake of Chinese residents was high, far exceeding the proposed intake for preventing non-communicable chronic disease (PI-NCD) by the WHO and dietary reference intakes (DRIs) in other countries, while the dietary intakes of K, Ca, Zn, Mg, and Se did not reach the PI-NCD and the DRIs in other countries. Intakes of P, Fe, and Mn did not meet the RDIs of some countries. Other nutrient elements were in the satisfactory intake range.

#### 3.2.1. Dietary Na and K Intake

The average dietary Na intake of Chinese residents was 4505 mg/d, which is about 3-times the AI value (1500 mg/d). The highest provincial intake was 4.3-times the AI value and the lowest was 1.9-times the AI value. This finding might be closely related to the fact that Chinese residents eat preserved foods and use too much salt for cooking. Excessive Na intake increases the risk of hypertension, and blood pressure in hypertensive patients is directly proportional to dietary Na intake.

The average dietary K intake of Chinese residents (2049 mg/d) reached 102% of the AI value (2000 mg/d). K intake among residents in 24 provinces ranged from 1004 mg/d (Ningxia) to 3412 mg/d (Beijing) and was insufficient in 13 provinces. The main dietary source of K was vegetables (40.8%), followed by cereals (18.2%). Dietary K intake was inversely related to the risk of developing blood pressure problems and the prevalence of hypertension. Dietary K counteracts the blood pressure-elevating effect of Na and helps to reduce the risk of hypertension [21].

**Figure 2:**
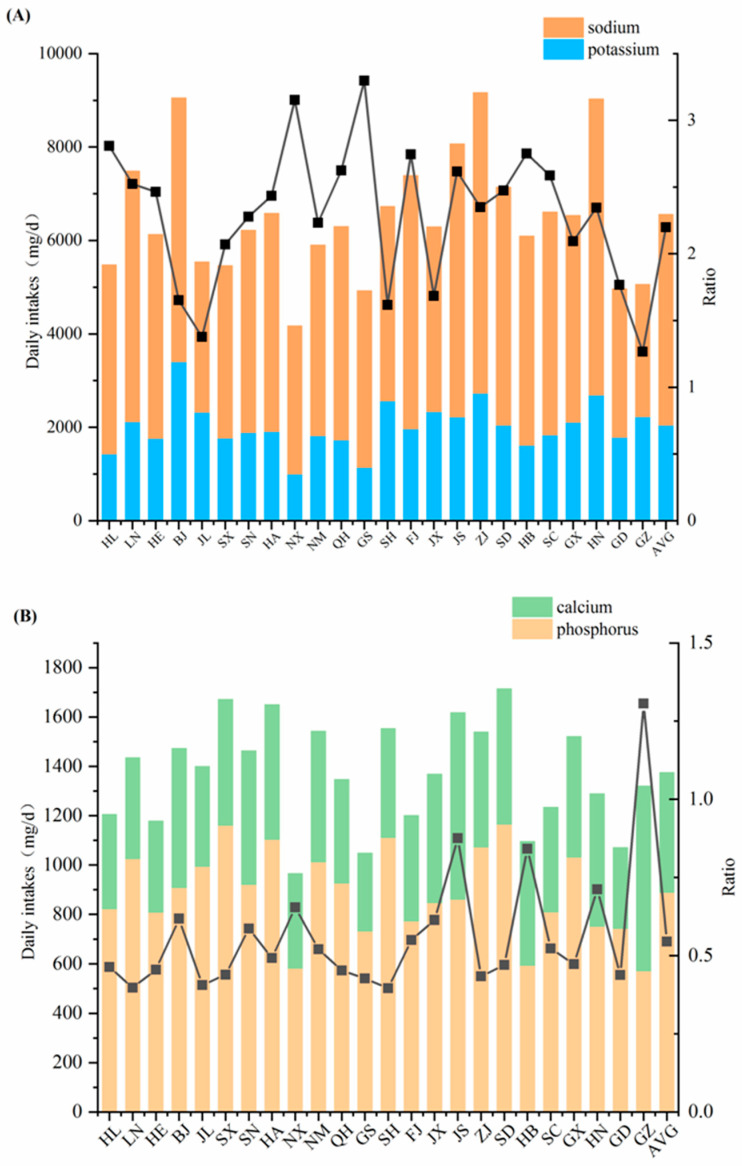
Dietary intake of K, Na, and Na/K ratio (**A**) and Ca, P, and Ca/P ratio (**B**) among participant regions in the 6th China TDS.

The dietary Na/K intake ratio of Chinese residents was between 1.3 and 3.3, and the average was 2.2. The ratios were relatively low in Guizhou and Jilin, at 1.3 and 1.4, respectively, mainly attributed to the low content of Na in dietary samples, especially in vegetables and cereals, which are the major food contributors to the Na and K intake. Gansu and Ningxia, on the contrary, showed the highest Na/K ratios at 3.3 and 3.2, since the contents of K in vegetable and cereal samples were apparently lower than the national average (Figure 2A). The appropriate ratio of dietary Na and K intake is 1:1, indicating an imbalance of the Na and K intake of most residents in China. It was recognized that the risk of hypertension increased by 0.341 times for every one-unit increase in the dietary Na/K ratio [28]. Residents are advised to incorporate more low-Na and high-K foods into their diet, including vegetables, fruits, liquid milk, unprocessed meat, and minimally processed grains. They should also reduce their consumption of foods that have a high Na/K ratio, such as cheese and butter, processed meat, soups, fast food, bread, and processed cereals. A reasonable dietary Na/K ratio can be achieved through the proper selection of food types.

#### 3.2.2. Dietary Ca and P Intake

The dietary Ca intake of Chinese residents ranged from 313 mg/d (Gansu) to 754 mg/d (Jiangsu). The dietary Ca intake in the 24 participant provinces did not reach the RNI value of Ca (800 mg/g), and the average level of 488 mg/d was only 61% of the Ca RNI (Figure 2B). The main sources of dietary Ca for Chinese residents included vegetables (32.0%), legumes (19.1%), and cereals (18.5%). That is, 70% of dietary Ca intake is derived from plant foods, and 19% is derived from animal foods. As a high-quality source of Ca, dairy contributes only 7.7% to dietary intake, which is directly related to the low consumption of dairy in the dietary structure of Chinese residents. The average dietary P intake of Chinese residents (890 mg/d) reached 124% of the P RNI value (RNI: 720 mg/d). It ranged from 572 mg/d (Guizhou) to 1166 mg/d (Shandong) among different provinces. Except for Ningxia, Hubei, and Guizhou, whose P intake was about 80% of the RNI, all the other participant provinces exceeded the RNI value. The main dietary sources of dietary P intake included cereals (38.4%), meat (15.7%), vegetables (13.7%), and legumes (12.5%), which together accounted for 80% of the total P intake.

The data obtained from the 6th TDS showed that the Ca/P ratio in the diet of Chinese residents was between 0.4 and 1.3, and the average ratio was 0.55. With the exception of Guizhou, all participant provinces had a Ca/P ratio of less than 1 (Figure 2B). Guizhou exhibited the highest Ca/P ratio. The Ca contents of vegetables and legumes in Guizhou were relatively high, almost two-times the national average, while the P content of cereals was the lowest among all the participant regions. An optimum dietary Ca/P intake ratio of between 1:1 and 2:1 is recommended for adequate absorption of Ca. As mentioned above, the dietary P intake level of Chinese residents basically satisfied the recommended amount, while Ca intake was seriously inadequate. It emphasized the importance of increasing Ca intake for Chinese residents in order to achieve a reasonable Ca/P ratio. Optimization of dietary patterns can be proposed to appropriately increase the consumption of legumes, dairy products, and aquatic foods, which are rich in Ca, as demonstrated in Table 1.

#### 3.2.3. Intake of Dietary Fe, Mn, Cu, Mo, and Cr

**Figure 3:**
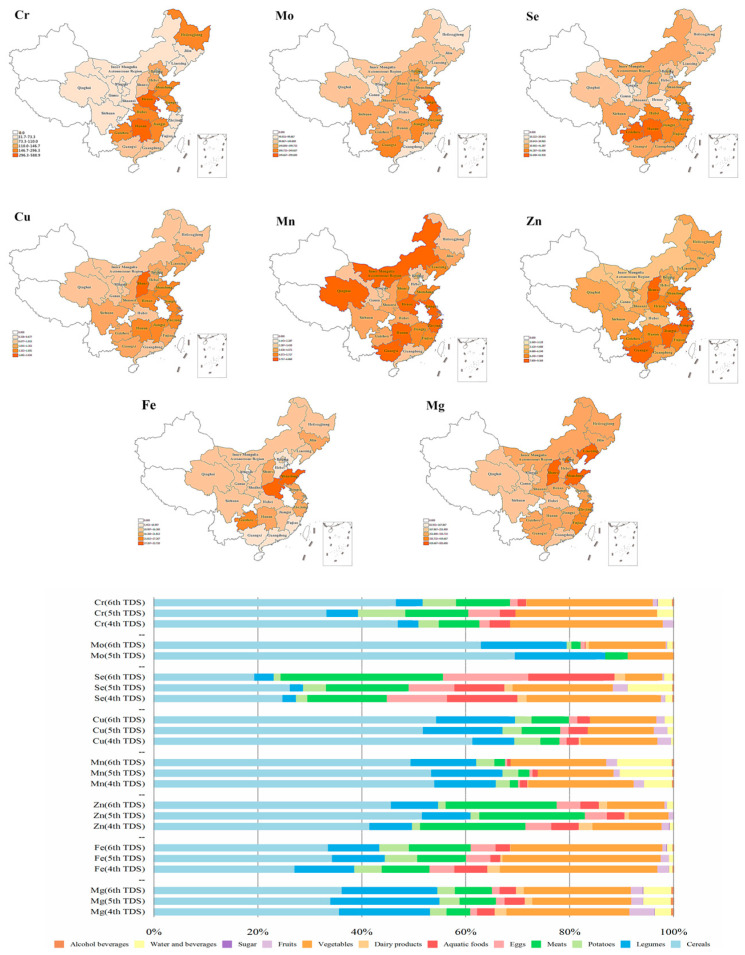
Regional distribution of estimated dietary intakes (EDIs) for nutrient elements among participant regions, and the percentage contribution of food categories to total EDIs. The upper figure represents the regional distribution of EDIs for nutrient elements, including Cr, Mo, Se, Cu, Mn, Zn, Fe, and Mg, respectively, in the 6th China TDS. The lower figure represents the percentage contribution of food categories to EDIs for Cr, Mo, Se, Cu, Mn, Zn, Fe, and Mg in the 4th, 5th, and 6th TDSs.

The average dietary Fe intake of Chinese residents (15.7 mg/d) reached 131% of the RNI value (12 mg/d). The residents in Hebei province had the lowest Fe intake (9.2 mg/d, 77% RNI), and those in Shandong province had the highest Fe intake (32.7 mg/d, 272% RNI) (Figure 3). The dietary Fe intake of the residents in seven provinces was below the RNI value, and Fe intake from animal foods in these provinces was mostly lower than the national average. With respect to the dietary Fe intake of Chinese residents, 75% came from plant-based diets (33.6% from cereals, 29.2% from vegetables, 9.9% from legumes, and 5.7% from potatoes), and 19.6% from animal-based diets (11.9% from meat, 4.8% from eggs, 2.8% from aquatic products, and 0.1% from dairy). The body’s absorption rate of Fe derived from plant foods is low, and the actual Fe intake of the residents may not meet the human body’s requirement for Fe. Therefore, it is necessary to include a greater proportion of animal foods in the residents’ diets in order to increase the body’s absorption of Fe.

The average dietary intake of Mn (4.8 mg/d) among Chinese residents reached 107% of the AI value (4.5 mg/d), ranging from 2.48 mg/d to 6.86 mg/d. The dietary Mn intake of residents in some regions was found to be inadequate, with the level of less than 75% AI in Gansu and Hebei provinces (Figure 3). Dietary intake of Mn is mainly derived from cereals, vegetables, and legumes, accounting for more than 80% of the total intake. Mn is implicated in the formation of bones and in the metabolism of carbohydrates, lipids, and amino acids. It is also involved in blood pressure, and brain function. Therefore, it is recommended to appropriately increase the intake of Mn-rich foods, such as legumes, in certain regions.

The average dietary Cu intake of Chinese residents (1.2 mg/d) reached 156% of the AI value (0.8 mg/d), ranging from 0.83 mg/d (104% AI, Ningxia) to 2.03 mg/d (254% AI, Shanxi) (Figure 3). The main sources included cereals, legumes, and vegetables, accounting for 82% of the total Cu intake.

The average dietary Mo intake of Chinese residents (161 μg/d) reached 161% of the RNI value (100 μg/d). The dietary Mo intake of residents in Gansu province was the lowest (91 μg/d), while dietary Mo intake among residents in Jiangsu province was the highest (299 μg/d) (Figure 3). The Mo intake of Chinese residents basically met the dietary intake requirements. Dietary Mo intake is mainly derived from cereals (63%), followed by legumes (14.8%) and vegetables (12.9%). Mo is ubiquitous in food and water and is easily absorbed. About 88–93% of soluble ammonium molybdate can be absorbed when orally ingested. At present, it has not been found that normal diets result in Mo deficiency [7,29].

The average dietary Cr intake of Chinese residents was 139.1 μg/d, ranging from 32.6 to 588.9 μg/d, all of which exceeded the AI value (30 μg/d). Dietary Cr intake and sources of dietary Cr showed significant regional differences. Dietary Cr intake was the highest in Henan province, where it was 19.6-times higher than the AI value, followed by Hunan province and Shandong province, which were 14.2- and 9.87-times higher than the AI value, respectively. The lowest Cr intake was observed in Shaanxi province, but it was also higher than AI (Figure 3), indicating a generally high Cr intake of Chinese residents. The main sources of Cr in the diet were cereals, vegetables, and meat, which contributed more than 80% to the total intake. Cereals were identified as the main source of dietary Cr intake, accounting for 46.65% of the total dietary sources, followed by vegetables and meat, accounting for 24.30% and 10.39%, respectively. The toxicity of Cr is highly dependent on its valence state. Cr mainly occurs in nature in two forms, namely trivalent and hexavalent, and there are obvious differences in the safety of the two valence states of Cr. Hexavalent Cr is more toxic than trivalent Cr. It is easily absorbed and poses a clear inhalational carcinogenic risk. As an essential trace element of the human body, the toxicity of trivalent Cr is very low, and it is implicated in certain physiological functions of the human body. Improper use of stainless-steel products for cooking and long-term storage of foods that contain high levels of acidity can cause Cr to dissolve. This may increase the Cr content of foods and result in excessive Cr intake. Future TDSs should detect the contents of different valences of elements in samples, so as to conduct more accurate dietary exposure and risk assessments.

#### 3.2.4. Dietary Intake of Mg, Zn, and Se

The average dietary Mg intake of Chinese residents (306 mg/d) was slightly higher than the RNI value (300 mg/d). The range was between 182 mg/d (Ningxia province) and 504 mg/d (Liaoning province), and the intake in 17 provinces was lower than the RNI value (Figure 3). The main dietary source of Mg intake was cereals (36.2%), followed by vegetables (20.6%) and legumes (18.4%). Mg deficiency increases the risk of high blood pressure and type 2 diabetes. The findings highlight the need to appropriately increase the consumption of Mg-rich legumes, whole grains, and vegetables.

The dietary Zn intake of Chinese residents was apparently lower than the RNI (12.5 mg/d), with an average level of 6.2 mg/d and ranging between 3.2 mg/d (Gansu) and 9.4 mg/d (Shanxi) (Figure 3). Cereals contributed the most (46%) to Zn intake, followed by meat (21%) and vegetables (11%). Low levels of consumption of Zn-rich foods, such as meat, eggs, and aquatic products, were observed among Chinese residents, resulting in a low Zn intake. Zn derived from different food types has different effects on metabolic syndrome, as well as cardiovascular and other diseases. Therefore, diets should be balanced and diversified, and Chinese residents should appropriately increase their intake of animal foods.

The average dietary Se intake of Chinese residents (36 μg/d) was only 59% of the RNI value (60 μg/d) and ranged from 11 (Ningxia province) to 62 μg/d (Hunan province). Of the 24 provinces, only Hunan (62 μg/d) reached the Se RNI value, and Guizhou (59 μg/d) was close to the RNI value (Figure 3). The average dietary Se intake (45 μg/d) of residents in the 12 provinces in the southern region was significantly higher than the average Se intake in the 12 provinces in the northern region (26 μg/d). The main source of dietary Se was meat (31.3%), followed by cereals (19.4%), aquatic products (16.6%), and eggs (16.4%). The results of the 6th TDS showed that the population in most regions of China was deficient in Se, to varying degrees. Se deficiency has been identified as a main risk factor of Kashin–Beck disease [30], while most recently, insufficient and unbalanced intakes of nutrients other than Se might be one of the main causes [31,32]. It affects the metabolism of lipids and carbohydrates and has a significantly negative correlation with hypertension. Therefore, emphasis should be placed on increasing the consumption of Se-rich foods to supplement the Se intake.

### 3.3. Comparison with Previous China TDSs and TDSs in Other Countries

**Figure 4:**
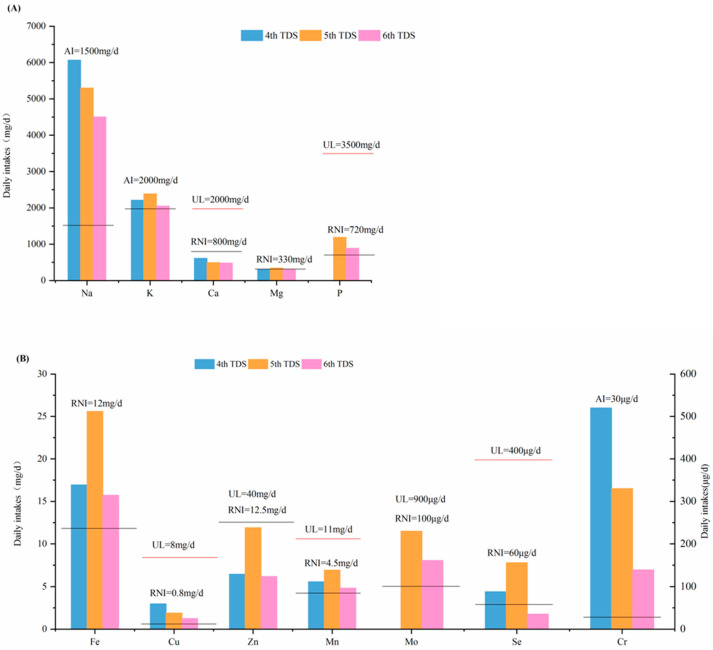
Dietary intake of macro-elements (**A**) and trace elements (**B**) from the 4th, 5th, and 6th TDSs, respectively. The red and black lines show the corresponding RNI and UL respectively.

Dietary nutrient intakes from three TDSs conducted in China were compared (Figure 4). In each of the TDSs, 12 food categories (i.e., cereal, legumes, potatoes, meat, eggs, aquatic products, dairies, vegetables, fruits, sugars, beverages and water, and alcohol) were investigated, covering more than 85% of the daily consumed food. In the 6th TDS, except for Mg, Fe, and Zn, the intakes of other macro-elements decreased from 8% (K) to 26% (Na), and the intakes of other trace elements decreased from 13% (Mn) to 77% (Se), when compared with the 4th and 5th TDSs. The intake of mineral nutrients such as Ca, Fe, Zn, and Se has declined, which is concerning and warrants attention. Both food consumption patterns and elemental content of food influenced the trends. The average daily food consumption increased from 1900 g/day in the 4th TDS, to 2759 g/day in the 5th TDS and 2649 g/day in the 6th TDS. Among the 12 food categories, the consumption of cereals significantly changed, with 400 g/day, 925 g/day, and 714 g/day in the 4th, 5th, and 6th TDSs, respectively. The variation in food consumption might result in the trends of some elements, such as K, Fe, Zn, Mn, and Se. For Na, Cu, and Cr, the contents in food played a more important role, leading to a significant decline in dietary intake over the last decade. Non-communicable diseases are on the rise globally. The “Salt Reduction Initiative in China (2010–2020 Action Plan)” and “Food and Nutrition Development in China (2014–2020)” listed salt control and low Na as goals. “Healthy China 2030” outlines an implementation target to reduce the national per capita daily salt intake by 20% by 2030. Compared with previous TDSs, the data from the 6th TDS showed a 26% decrease in Na intake compared to the data obtained from the 4th TDS (6068 mg/d). Moreover, the Na intake in the 6th TDS was 15% lower than that outlined in the 5th TDS (5302 mg/d). Dietary Na intake continued to decline. The dietary Na intake of residents in the 24 provinces (municipalities and autonomous regions) exceeded the AI value (1500 mg/d), as well as the maximum intake of Na for adults recommended by the WHO (2 g/d) [22]. Therefore, it is necessary to carry out continuous salt reduction actions. Residents should reduce their intake of salt and condiments in order to reduce their dietary intake of Na [33], which can help to decrease the risk of coronary heart disease, hypertension, and cardiovascular diseases [22].

The macro-element and trace element intakes of Chinese residents, as outlined in the 6th TDS, were compared with TDSs conducted in other countries and regions (Table 3). The dietary Na and Mn intakes of Chinese residents were higher, dietary Fe and Mg intakes were slightly higher than those of other countries/regions, while dietary K, Ca, P, Cu, Zn, and Se intakes were relatively low [34,35,36,37,38,39,40,41,42,43,44,45]. This might be due to the low levels of consumption of meat, eggs, aquatic products, and other foods in the diet structure of Chinese residents. The data obtained from the 4th and 5th TDSs showed that dietary Cr exposure in China was higher than that in France. However, according to the 6th TDS, dietary Cr intake in China was only 50% of the dietary intake of French residents [34]. The main sources of dietary nutrient intakes in China are not very different to those observed in other countries and regions. In Australia [40], France [34], the United Kingdom [38], and Hong Kong of China [36], cereals are the main source of dietary Mo intake. In France [34], Australia [40], the United States [37], and Hong Kong of China [36], cereals are the main sources of dietary Fe intake. In France [34], the United States [37], and Hong Kong of China [36], cereals and vegetables are the main sources of dietary Mg intake. In France [34], Australia [40], the United States [37], and Hong Kong of China [36], vegetables are the main sources of dietary K intake. In Australia [40], Ireland [39], New Zealand [43], the United Kingdom [38], and Hong Kong of China [36], cereals, meat, and aquatic products are the main sources of dietary Se intake. Cereals and meat are the main sources of dietary Zn intake in Australia [40], France [34], the United Kingdom [38], and Hong Kong of China [36]. In the United States [37] and France [34], meat and cereals are the main sources of dietary Na intake, probably because residents add various sauces with a high Na content during the cooking process. Among mainland Chinese residents, plant foods are the main source of P, and the intake of foods with a high P content, such as meat, eggs, and aquatic products, is lower than that of Hong Kong [36] and the United States [37]. The comparative data revealed that, among Chinese residents, the main dietary sources of most nutrients are similar to those of other countries and regions. However, it is notable that these TDSs were conducted at different times in different countries, using different research methods, data collection methods, and experimental methods of sample analysis. They might also differ in their approach to processing the results that fell below the limit of detection.

## 4. Conclusions

The 6th China TDS involved a quantitative investigation of nutrient elements in 12 food categories from 24 provinces (municipalities and autonomous regions), analyzing their concentration and distribution characteristics, dietary exposure, and potential health effects. It is a most recent, large-scale, and reliable study on the nutrient elements in the Chinese diet, representing the dietary habits in various regions of China and covering more than two-thirds of the total population. Trace elements in food were found to be compatible with their abundance in the earth’s crust. Plant foods were the main source of both macro-elements and trace elements for the Chinese population, accounting for about 68–96% of the total intake, while animal foods accounted for only 3–31%. The average dietary Na intake of Chinese residents was still high, which produced an unsatisfactory dietary Na/K ratio. Average dietary intakes of K, P, Mn, Fe, Cu, Mo, and Cr met the health guideline values. The average intake of dietary Ca, Mg, Zn, and Se has not yet reached the recommended value, and Ca deficiency is still a common nutritional problem, resulting in a serious imbalance in the Ca/P ratio. This finding is probably related to the low proportion of animal food in the overall diet of Chinese residents. The findings indicate that the current dietary structure of Chinese residents is still imbalanced, although it has gradually improved compared with the results from the previous China TDSs. In this regard, it is necessary to promote dietary guidance and optimize the dietary structure, while continuing to implement salt reduction measures, so as to encourage healthier dietary patterns and achieve balanced nutrient intake among the population.

## Figures and Tables

**Table 1:** Average concentrations of nutrient elements in 12 food categories in the 6th China TDS. The color shows relative abundance among different food categories, with the dark color indicating a higher concentration and the light color indicating a lower value. P, Na, K, Ca, Mg in mg/g; Mn, Fe, Zn, Cu in mg/kg; Se, Mo, Cr in μg/kg.

Food Categories	Nutrient Elements											
	P	Na	K	Ca	Mg	Mn	Fe	Zn	Cu	Se	Mo	Cr
Cereals	0.48	1.14	0.53	0.13	0.15	3.33	6.77	3.92	0.94	10.1	143.7	86.8
Legumes	1.63	3.78	2.48	1.40	0.79	8.74	22.27	7.96	2.68	19.3	379.3	103.1
Potatoes	0.33	4.47	2.04	0.14	0.16	2.95	12.64	1.52	0.66	6.7	22.4	129.6
Meats	1.65	8.11	2.62	0.26	0.25	1.33	19.89	15.60	1.03	128.0	33.3	140.6
Eggs	2.21	7.48	1.50	0.54	0.17	0.67	28.10	10.86	0.76	230.0	53.0	77.0
Aquatic foods	1.78	6.70	2.82	0.75	0.33	1.15	16.08	8.61	1.04	214.9	7.5	81.1
Dairy products	1.13	0.36	1.72	1.24	0.15	0.04	0.77	3.53	0.04	25.9	36.6	4.9
Vegetables	0.34	5.59	2.33	0.43	0.17	2.47	12.52	1.90	0.46	7.0	66.0	90.5
Fruits	0.12	0.01	1.59	0.08	0.13	1.76	2.47	0.46	0.44	2.6	6.3	30.9
Sugar	0.07	0.08	0.75	0.15	0.12	0.58	4.37	0.43	0.27	3.1	2.9	55.9
Water and beverages	0.008	0.02	0.05	0.04	0.02	0.63	0.18	0.08	0.02	0.5	1.7	3.7
Alcohol	0.07	0.02	0.16	0.04	0.04	0.24	0.38	0.10	0.04	1.2	3.4	9.5

**Table 2:** Dietary intake of nutrient elements from the 6th China TDS.

Category	Na (mg/d)	K (mg/d)	Ca (mg/d)	Mg (mg/d)	P (mg/d)	Mn (mg/d)	Fe (mg/d)	Zn (mg/d)	Cu (mg/d)	Se (μg/d)	Mo (μg/d)	Cr (μg/d)
Cereals	812.6	374.0	91.2	111.6	346.6	2.39	5.27	2.82	0.68	6.9	101.6	64.9
Legumes	279.0	171.0	90.6	56.4	110.5	0.61	1.56	0.56	0.19	1.3	26.6	7.2
Potatoes	324.1	131.1	9.4	10.0	22.5	0.17	0.89	0.09	0.04	0.5	1.5	8.9
Meats	672.2	224.6	19.2	21.5	137.8	0.10	1.87	1.32	0.09	11.2	2.9	14.5
Eggs	198.1	39.8	14.2	4.6	57.6	0.02	0.75	0.28	0.02	5.8	1.3	2.1
Aquatic foods	198.3	75.2	21.4	9.5	47.1	0.03	0.44	0.22	0.03	5.9	0.2	2.3
Dairy products	10.9	50.9	36.3	4.5	32.8	0.00	0.01	0.10	0.00	0.7	1.1	0.1
Vegetables	1969.1	823.0	153.2	62.2	119.4	0.89	4.59	0.68	0.16	2.6	23.8	33.8
Fruits	0.8	83.9	3.9	6.9	6.8	0.10	0.12	0.03	0.02	0.1	0.4	1.1
Sugar	0.1	1.6	0.3	0.4	0.5	0.00	0.02	0.00	0.00	0.0	0.0	0.2
Water and beverages	19.4	44.3	40.3	16.2	6.3	0.51	0.17	0.07	0.02	0.6	1.8	3.8
Alcohol	0.6	4.0	0.7	0.9	1.6	0.01	0.01	0.00	0.00	0.0	0.1	0.2
SUM	4485.3	2023.5	480.8	304.9	889.5	4.83	15.72	6.17	1.25	35.7	161.1	139.1
AI	1500	2000	–	–	–	–	–	–	–	–	–	30
RNI	–	–	800	330	720	4.5	12	12.5	0.8	60	100	–
UL	–	–	2000	–	3500	11	42	40	8	400	900	–

Note: the AI, RNI, and UL values were from the “Chinese Dietary Reference Intakes (2013 version)”.

**Table 3:** Summary of dietary intake of nutrient elements presented in the different TDSs.

Country/ Region	Na (g/d)	K (g/d)	Ca (mg/d)	Mg (mg/d)	P (mg/d)	Fe (mg/d)	Mn (mg/d)	Cu (μg/d)	Mo (μg/d)	Se (μg/d)	Zn (mg/d)	Cr (μg/d)	Reference
USA	1.7–2.7	1.9–2.7	510–800	180–260	890–1400	9.0–14	1.9–2.9	730–1400	-	66–130	7.6–13	-	[37]
France	2.65	2.854	786	304	-	7.71	2.16	1940	93.9	64.4	7.93	277	[34]
Australia	-	3.0–4.4	700–1200	-	-	8.7–15	3.9–5.4	1200–1900	76–120	97–170	7.8–15	-	[40]
Canada	-	-	-	-	-	-	2.6–3.8	900–1500	-	110–220	7.2–16	-	[42]
UK	-	-	-	-	-	-	3.9–5.5	1100–1300	98–150	45–67	6.5–9.8	-	[38]
Ireland	-	-	-	-	-	-	-	-	-	58.3–68.4	-	51–89	[39]
New Zealand	2.899	-	-	-	-	-	-	-	-	83	12.7	-	[43]
Lebanon	-	-	-	-	-	13.0	2.04	1104.19	-	-	10.97	-	[44]
Brazil	1.93	0.861	275	-	-	5.7	-	-	-	-	4.25	20.7	[35]
Italy	3.81	2.91	738	262	-	11–12.7	-	1.14	-	-	10.6–12	-	[45]
Cameroon	2.1	2.1	760	294	-	6.67	-	-	-	-	6.54	-	[41]
China/Hong Kong	2.6	1.9	430	210	1000	8	4.4	920	110	140	9.2	-	[36]
China	4.5	2.05	485	306	890	15.7	4.83	1250	161	35.69	6.2	139.1	present study

Note: all the data (means or ranges) are in the same format as in the original literature.

## Data Availability

Data are contained within the article.

## Supplementary Materials

The following supporting information can be downloaded at: https://www.mdpi.com/article/10.3390/nu15112613/s1, Figure S1: Regional differences in elemental content of cereals (Na, K, P, Ca, Mg, Fe), legumes (Cr), and vegetables (Cr). Table S1: International and national guidance values for the intake of nutrient elements. Table S2: Contents of elements in standard reference materials for method validation and quality control (mg/kg). Table S3: Concentrations of nutrient elements in diet samples from the 6th China TDS.
