# Supplementary material for: Dietary Intake of Multiple Nutrient Elements and Associated Health Effects in the Chinese General Population from a Total Diet Study
Source: Nutrients. 2023 Jun 2;15(11):2613. doi: 10.3390/nu15112613 (PMC10255428; doi:10.3390/nu15112613)
Supplement: Supplementary file 1 [file nutrients-15-02613-s001.zip › nutrients-2374999-supplementary.pdf]

Dietary Intake of Multiple Nutrient Elements and Associated Health Effects in the Chinese General Population from a Total Diet Study

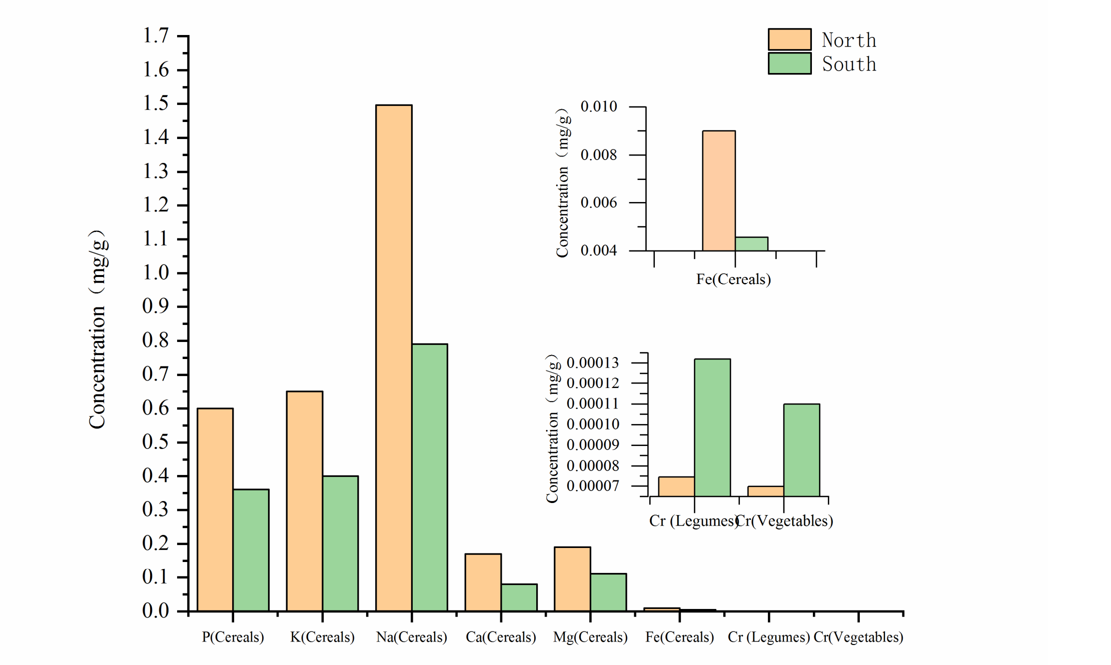

Figure S1. Regional differences in elemental content of cereals (Na, K, P, Ca, Mg, Fe), legumes (Cr), and vegetables (Cr).

Table S1. International and national guidance values for the intake of nutrient elements.

	WHO (WHO, 2012a; WHO, 2012b)	Australia and New Zealand (NHMRC, 2017)		Canada (HC, 2010)		US (FNB, 2019)		Cuban (INHA and the Cuban Ministry of Public Health, 2008)		Oman (Alasfoor et al., 2009)
	PI–NCD	RDI/ AI	UL	RDA/AI	UL	RDA/AI	UL	DRI/AI	UL	RNI
Sodium	2000 mg/d	*460–920 mg/d	ND	*1500 mg/d	2300 mg/d	*1500 mg/d	ND	500 mg/d	2.3 g/d	5.3–6.0 g/d
Potassium	3510 mg/d	*2800 mg/d	NP	*4700 mg/d	ND	*3400 mg/d	ND	2000 mg/d	3500 mg/d	–
Calcium	–	1000 mg/d	2500 mg/d	1000 mg/d	2500 mg/d	1000 mg/d	2500 mg/d	*800 mg/d	2500 mg/d	525–960 mg/d
Phosphorus	–	1000 mg/d	4000 mg/d	700 mg/d	4000 mg/d	700 mg/d	4 g/d	800 mg/d	4000 mg/d	–
Magnesium	–	310–320 mg/d	350 mg/d	400–420 mg/d	350 mg/d	400–420 mg/d	350 mg/d	325 mg/d	350 mg/d	–
Iron	–	18 mg/d	45 mg/d	8 mg/d	45 mg/d	8 mg/d	45 mg/d	17 mg/d	45 mg/d	11.6–13.2 mg/d
Selenium	–	60 μg/d	400 μg/d	55 μg/d	400 μg/d	55 μg/d	400 μg/d	55 μg/d	400 μg/d	–
Manganese	–	*5.0 mg/d	NP	*2.3 mg/d	11 mg/d	*2.3 mg/d	11 mg/d	*2 mg/d	11 mg/d	–
Zinc	–	8 mg/d	40 mg/d	11 mg/d	40 mg/d	11 mg/d	40 mg/d	12 mg/d	40 mg/d	21–24 mg/d
Copper	–	*1.2 mg/d	10 mg/d	900 μg/d	10000 μg/d	900 μg/d	10000 μg/d	900 μg/d	10000 μg/d	–
Iodine	–	150 μg/d	1100 μg/d	150 μg/d	1100 μg/d	150 μg/d	1100 μg/d	150 μg/d	1100 ug/d	158–180 μg/d
Molybdenum	–	45 μg/d	2000 μg/d	45 μg/d	2000 μg/d	45 μg/d	2000 μg/d	45 μg/d	1700–2000 μg/d	–
Chromium	–	*25 μg/d	NP	*35 μg/d	ND	*35 mg/d	ND	*35 μg/d	ND	–

*Adequate Intakes (AIs) in ordinary type.

NP, not possible to set may be insufficient evidence or no clear level for adverse effects; ND, not determined – reflecting the inability to identify a single point below which there is low risk.

Table S2. Contents of element in standard reference materials for method validation and quality control (mg/kg).

standard reference materials		Na	K	Ca	Mg	P	Mn	Fe	Zn	Cu	Se	Mo
SRM 1568b	certified value	6.74±0.19	1282±11	118.4±3.1	559±10	1530±40	19.2±1.8	7.42±0.44	19.42±0.26	2.35±0.16	0.365±0.029	1.451±0.048
	determined value	6.57	1273	120	553	1543	19.2	7.56	18.63	2.4	0.341	1.412
SRM 1570a	certified value	1.818 ±0.043%	2.903± 0.052%	1.527± 0.041%	—	0.518±0.011%	75.9±1.9	—	82±3	12.2±0.6	0.117±0.009	—
	determined value	1.80%	2.881%	1.546%	—	0.511%	77.1	—	84	12.3	0.119	—
SRM 1577c	certified value	0.2033± 0.0064%	1.023±0.064%	131±10	620±42	—	10.46±0.47	197.94±0.65	181.1±1.0	275.2±4.6	2.031±0.045	3.30±0.13
	determined value	0.20%	1.01%	134	604	—	10.33	197.51	179	275.8	1.996	3.21
ERM-BD150	certified value	4.18±0.19 g/kg	17.0±0.7 g/kg	13.9±0.8 g/kg	1.26±0.10 g/kg	11.0±0.6 g/kg	0.289±0.018	4.6±0.5	44.8±2.0	1.08±0.06	0.188±0.014	—
	determined value	4.26 g/kg	16.4 g/kg	14.3 g/kg	1.29 g/kg	11.1 g/kg	0.31	4.4	43.6	1.04	0.181	—
ERM-BD151	certified value	4.19±0.23 g/kg	17.0±0.8 g/kg	13.9±0.7 g/kg	1.26±0.07g/kg	11.0±0.6 g/kg	0.29±0.03	53±4	44.9±2.3	5.00±0.23	0.19±0.04	—
	determined value	4.37 g/kg	16.9 g/kg	13.6 g/kg	1.30 g/kg	10.8 g/kg	0.28	50	47.0	4.81	0.16	–
SRM 1566b	certified value	0.3297±0.0053%	0.652±0.009%	0.0838±0.0020%	0.1085±0.0023%	—	18.5±0.2	205.8±6.8	1424±46	71.6±1.6	2.06±0.15	—
	determined value	0.33%	0.64%	0.02%	0.109%	–	18.6	211.6	1436	72.6	1.96	—

Table S3. Concentrations of nutrient elements in diet samples from the 6th China TDS.

Category	Mean (Range)											
	Na (mg/g)	K (mg/g)	Ca (mg/g)	Mg (mg/g)	P (mg/g)	Mn (mg/kg)	Fe (mg/kg)	Zn (mg/kg)	Cu (mg/kg)	Se (μg/kg)	Mo (μg/kg)	Cr (μg/kg)
Cereals	1.14 (0.44–2.21)	0.53 (0.31–0.92)	0.13 (0.04–0.24)	0.15 (0.07–0.29)	0.48 (0.20–0.75)	3.33 (2.11–4.49)	6.77 (0.69–3.89)	3.92 (2.47–5.96)	0.94 (0.67–1.52)	10.1 (0.2–35.4)	143.7 (91.8–245.6)	86.8 (4.7–430.8)
Legumes	3.78 (1.89–7.10)	2.48 (1.13–4.67)	1.40 (0.51–2.72)	0.79 (0.39–1.69)	1.63 (0.95–2.21)	8.74 (3.71–15.1)	22.27 (11.77–41.31)	7.96 (3.52–13.06)	2.68 (1.34–3.58)	19.3 (0.1–65.0)	379.3 (142.9–680.8)	103.1 (22.6–667.9)
Potatoes	4.47 (0.60–8.38)	2.04 (0.60–3.43)	0.14 (0.08–0.30)	0.16 (0.05–0.39)	0.33 (0.21–0.52)	2.95 (0.59–8.59)	12.64 (5.29–27.63)	1.52 (0.42–3.61)	0.66 (0.25–1.41)	6.7 (ND–22.7)	22.4 (ND–57.6)	129.6 (19.9–756.3)
Meats	8.11 (5.69–11.25)	2.62 (1.51–4.48)	0.26 (0.06–1.47)	0.25 (0.12–0.39)	1.65 (1.09–2.26)	1.33 (0.24–2.76)	19.89 (8.97–36.67)	15.60 (10.63–27.07)	1.03 (0.47–2.31)	128.0 (82.5–186.4)	33.3 (6.4–74.2)	140.6 (28.3–856.0)
Eggs	7.48 (1.45–14.78)	1.50 (0.99–2.56)	0.54 (0.33–0.71)	0.17 (0.10–0.23)	2.21 (1.15–2.84)	0.67 (0.29–1.67)	21.80 (15.90–44.01)	10.86 (7.58–14.00)	0.76 (0.46–1.03)	230.0 (118.0–410.2)	53.0 (32.8–126.0)	77.0 (0.7–381.6)
Aquatic foods	6.70 (3.50–10.96)	2.82 (2.16–3.94)	0.75 (0.25–1.94)	0.33 (0.13–0.53)	1.78 (1.08–3.03)	1.15 (0.16–2.71)	16.08 (5.28–45.39)	8.61 (4.48–22.51)	1.04 (0.24–3.65)	214.9 (109.0–336.2)	7.5 (ND–24.9)	81.1 (11.2–264.3)
Dairy products	0.36 (0.28–0.52)	1.72 (1.32–2.27)	1.24 (0.91–1.84)	0.15 (0.11–0.29)	1.13 (0.78–1.67)	0.04 (0.02–0.08)	0.77 (0.09–7.58)	3.53 (2.65–5.01)	0.04 (0.02–0.07)	25.9 (13.3–51.0)	36.6 (27.4–48.0)	4.9 (0.5–27.0)
Vegetables	5.59 (3.27–7.61)	2.33 (1.57–3.82)	0.43 (0.28–0.82)	0.17 (0.09–0.29)	0.34 (0.23–0.51)	2.47 (1.41–4.51)	12.52 (4.68–39.40)	1.90 (1.25–3.25)	0.46 (0.28–0.79)	7.0 (0.6–18.6)	66.0 (18.9–378.5)	90.5 (20.6–292.8)
Fruits	0.01 (0.00–0.03)	1.59 (1.08–1.99)	0.08 (0.03–0.33)	0.13 (0.08–0.18)	0.12 (0.07–0.17)	1.76 (0.41–6.82)	2.47 (0.88–8.75)	0.46 (0.19–2.05)	0.44 (0.30–0.64)	2.6 (0.1–10.1)	6.3 (0.3–17.9)	30.9 (2.4–316.0)
Sugar	0.08 (0.00–0.47)	0.75 (0.01–4.15)	0.15 (0.01–0.95)	0.12 (0.00–1.04)	0.07 (0.00–1.56)	0.58 (0.03–4.71)	4.37 (0.00–51.58)	0.43 (ND–7.39)	0.27 (0.00–5.09)	3.1 (0.1–21.1)	2.9 (0.3–41.98)	55.9 (0.5–585.7)
Water & beverages	0.02 (0.01–0.05)	0.05 (0.00–0.24)	0.04 (0.01–0.07)	0.02 (0.00–0.05)	0.008 (0.00–0.04)	0.63 (0.00–4.04)	0.18 (0.00–0.61)	0.08 (0.007–0.25)	0.02 (0.00–0.11)	0.5 （0.1–1.6）	1.7 (0.3–4.7)	3.7 (0.5–43.8)
Alcohol beverages	0.02 (0.00–0.07)	0.16 (0.00–0.34)	0.04 (0.00–0.33)	0.04 (0.00–0.08)	0.07 (0.00–0.16)	0.24 (0.00–1.29)	0.38 (0.03–1.84）	0.10 (0.02–2.22)	0.04 (0.00–0.17)	1.2 （0.1–2.2）	3.4 (0.3–25.9)	9.5 (0.5–108.3)

Note: values not detected（ND）were treated as being equal to half of the limit of detection (LOD) while calculating the mean value.
